# The Best Alkaline Wash

**Published:** 1901-12

**Authors:** W. Harpur Sloan

**Affiliations:** Philadelphia, Pa., Chief of Ear Department, Medico-Chirurgical College


### CLINICAL REPORTS.

### The Best Alkaline Wash.

BY W. HARPUR SLOAN, M. D., Philadelphia, Pa.,
Chief of Ear Department, Medico-Chirurgical College.

There are many alkaline preparations on the market that are used daily with varied results in conditions where such a preparation is indicated. I have tried most of them in all conditions and after an impartial trial, I am compelled to say that the preparation known as glyco-thymoline, made by Kress & Owen Co., stands at the head of the list; its formula is one that would commend its use, the ingredients being of an antiseptic and non-irritating nature.

Having formed this opinion of glyco-thymoline, I submit a report of a few clinical cases where it has given me good results.

Case I.—M.L., aged 26 years, came under my care suffering with a distressing case of ozena. The turbinated bones on both sides of her nose presented a condition of marked atrophy; there was a complete loss of smell and taste, and a formation of crusts in the nasal chamber, and stench of same was foul. She complained of continual headache and other symptoms of a depleted and run down system. I placed her on a tonic of iron, arsenic and strychnia, internally; locally, I ordered the use of glyco-thymoline in a Bermingham douche, three times a day, diluted. After a month's treatment the crusts had ceased to form; there was a complete restoration of taste and a slight return of smell; the general health was improved, and the patient herself well satisfied with results.

Case II.—C. A., aged 8 years, came to me suffering with a severe otorrhea following scarlet fever. There was a muco-purulent discharge from both ears that rendered the child completely deaf; the auditory canal was excoriated and sore, and the general health below par. I used cod liver oil internally, and syringed the ears three times a day with glyco-thymoline. At the end of one month the discharge of pus had stopped; the hearing much improved, and the child's general health very much better.

Case III.—J. W., aged 25 years, came under my care suffering with an aggravated case of cystitis, which had been treated by several of our best physicians, without much improvement. He had great pain in the region of the bladder and loins, which became worse on urination; a heavy deposit of mucus and some blood in the urine made his condition more distressing; his temperature was 100°, which would rise a degree during the periods of pain. I used the usual treatment for such cases without positive results, when I thought of irrigating the bladder with glyco-thymoline (dilute). This I did once in the twenty-four hours, at the same time giving him glyco-thymoline internally in teaspoonful doses every three hours. For the first two days I did not see much improvement; on the third day there was no blood in the urine and less mucus. I continued this treatment for two weeks, when I discharged him cured.

Case IV.—J. H., aged 35 years, consulted me for pruritus ani which had troubled him for several years; his business compelled him to sit the best part of the day. He had used various ointments, prescriptions, and the like, for this troublesome affection, with only temporary relief. At his first visit I ordered him to bathe the rectum twice daily with castile soap and warm water, then to apply glyco-thymoline, half strength, to the parts. After persisting for a time, the swelling and severe itching was lessened, and soon ceased altogether.

